# Ulnar bone tuberculosis in children: Case report and literature review

**DOI:** 10.1097/MD.0000000000038611

**Published:** 2024-06-21

**Authors:** Qineng Mo, Xiaohua Wei, Xiansheng Xia, Yunlong Li, Guoxin Nan, Chunli Ling

**Affiliations:** a Pediatric Orthopedics, Dongguan Children’s Hospital Affiliated to Guangdong Medical University, Dongguan City, China; b Osteoarthritis, Dongguan Children’s Hospital Affiliated to Guangdong Medical University, Dongguan City, China.

**Keywords:** bone tuberculosis, pathology, swelling, ulna

## Abstract

**Rationale:**

Tuberculosis of the long tubular bones in children’s extremities is infrequent, particularly in the ulna. Early diagnosis poses significant challenges. This report presents a case involving a 2-year-old child with tuberculosis of the ulnar bone, accompanied by a comprehensive review of pertinent literature. The purpose of this study is to share diagnostic and therapeutic experiences and provide potentially valuable insights.

**Patient concerns:**

In this case, the patient exhibited complete destruction and expansion of the ulnar bone, resulting in a forearm size considerably greater than normal. Concerns were raised about the irreversible deformation of the ulna, the potential for a malignant bone tumor, and its impact on forearm function, potentially endangering the patient’s life.

**Diagnoses:**

The diagnosis was confirmed as tuberculosis of the ulnar bone.

**Interventions:**

The patient underwent surgery to remove the affected ulnar tissue and received anti-tuberculosis medication.

**Outcomes:**

Subsequent to treatment, the destruction and expansion of the ulnar bone resolved, with the return of normal ulnar morphology and bone structure.

**Lessons:**

Even in the absence of typical symptoms like fever, weight loss, and loss of appetite, extensive destruction and expansion of a long tubular bone should prompt vigilant consideration of bone tuberculosis.

## 1. Introduction

Bone tuberculosis, or osteoarticular tuberculosis, is an infectious condition where Mycobacterium tuberculosis affects bones or joints. Though clinically uncommon, its incidence is even less frequent in long tubular bones compared to spinal or other bone and joint tuberculosis. It often gets misidentified as chronic suppurative osteomyelitis, Langerhans cell histiocytosis, bone fibrous dysplasia, bone cysts, or malignant tumors, causing treatment delays. Our hospital recently treated a 2-year-old with ulnar bone tuberculosis (UBT), we have reported on it and introduced the treatment and prognosis.

## 2. Case presentation

**Figure 1.**
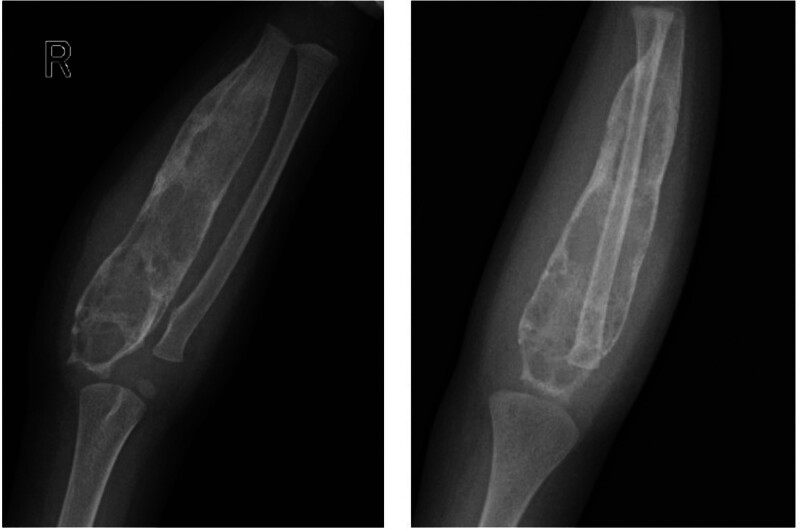
Anteroposterior and lateral X-ray of the ulna depicted the progression of bone expansion in the right ulna, including lateral cortical thinning and disorganized trabecular bone structure, accompanied by high-density shadows indicative of cord-like, patchy, and ground-glass appearances.

**Figure 2.**
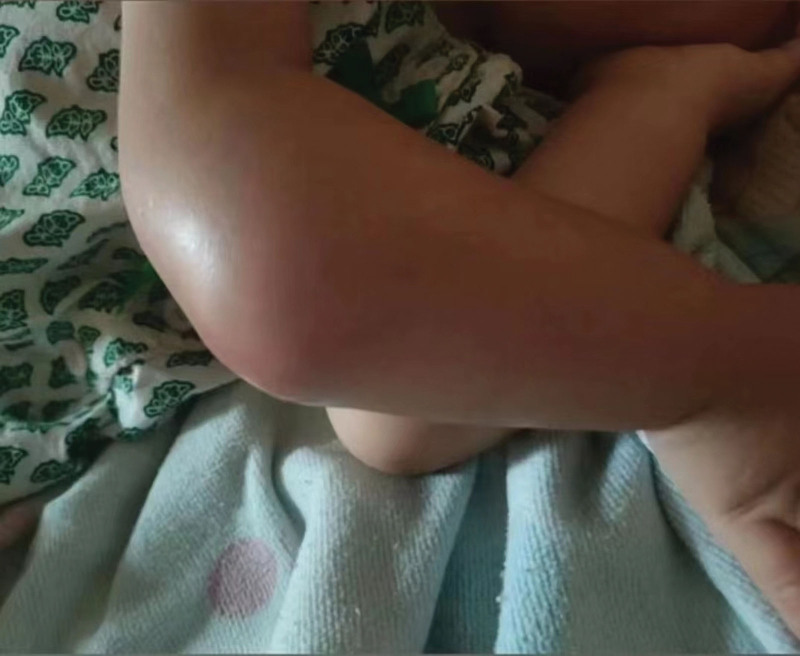
Deformed right forearm with noticeable swelling at the ulna’s proximal end and a fluctuant cold swelling.

**Figure 3.**
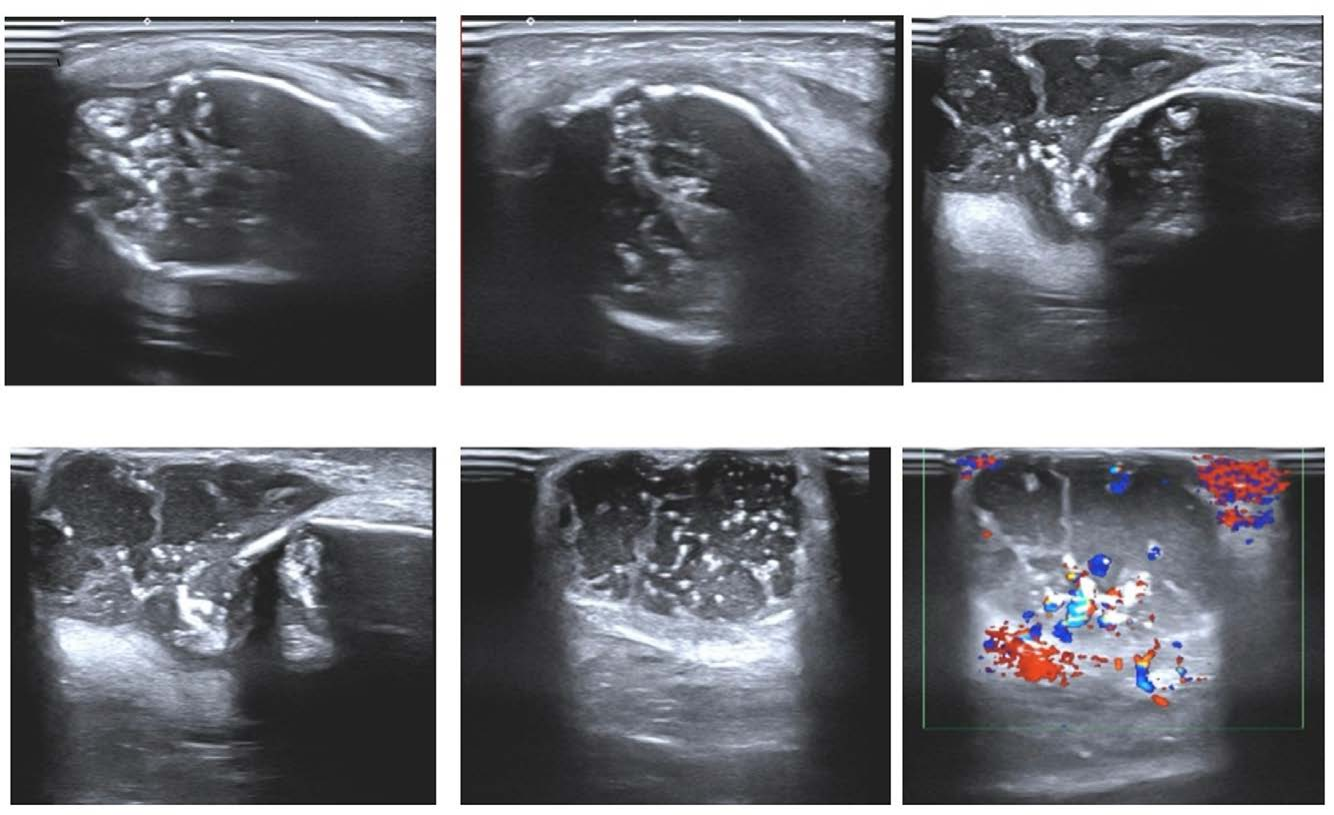
The right ulna underwent expansive changes, characterized by thinning of the lateral cortex, disrupted echogenicity, and extension into soft tissue, resulting in a cystic mass with mixed echogenicity in the soft tissue layer, measuring approximately 36 × 22 mm, surrounded by significant blood flow signals on color Doppler flow imaging (CDFI).

**Figure 4.**
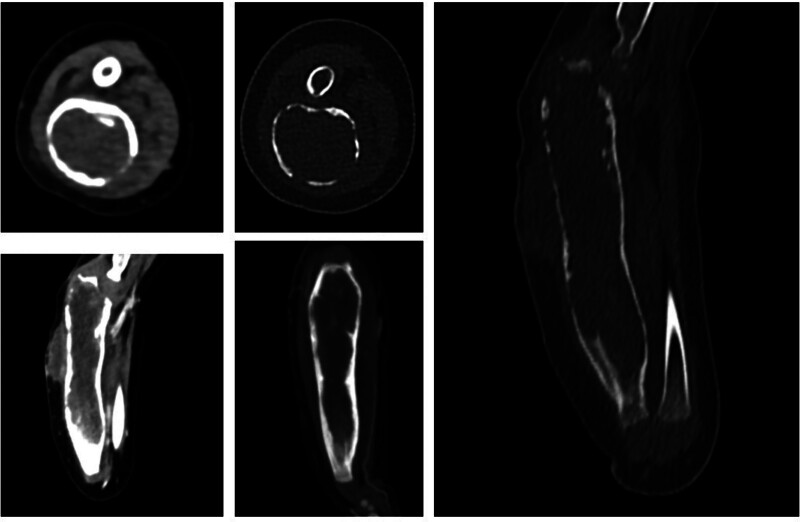
The entire length of the right ulna exhibited expansive bone destruction, approximately 93 mm in length, with thinning and multiple areas of cortical disruption and irregular density within the bone marrow cavity. Contrast-enhanced imaging revealed a cord-like, patchy, and uneven soft tissue mass associated with local bone destruction, penetrating the cortical bone.

**Figure 5.**
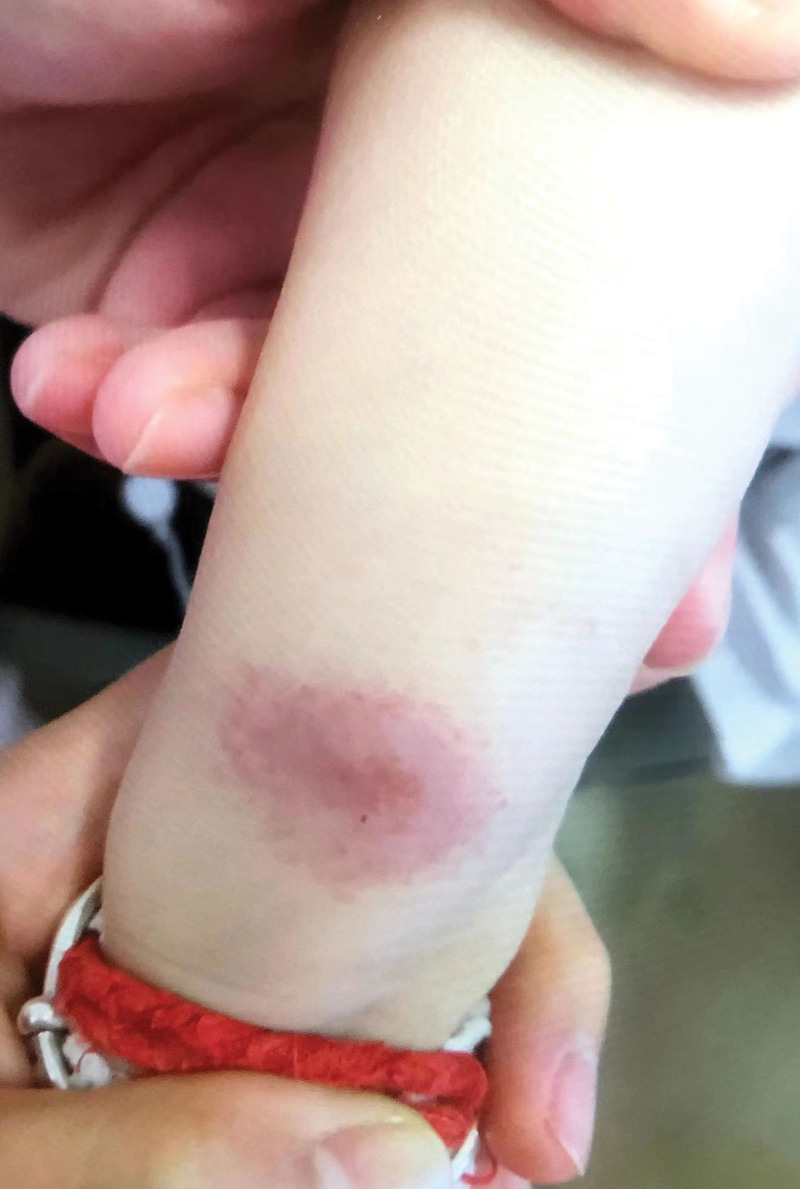
The tuberculin test was strongly positive.

**Figure 6.**
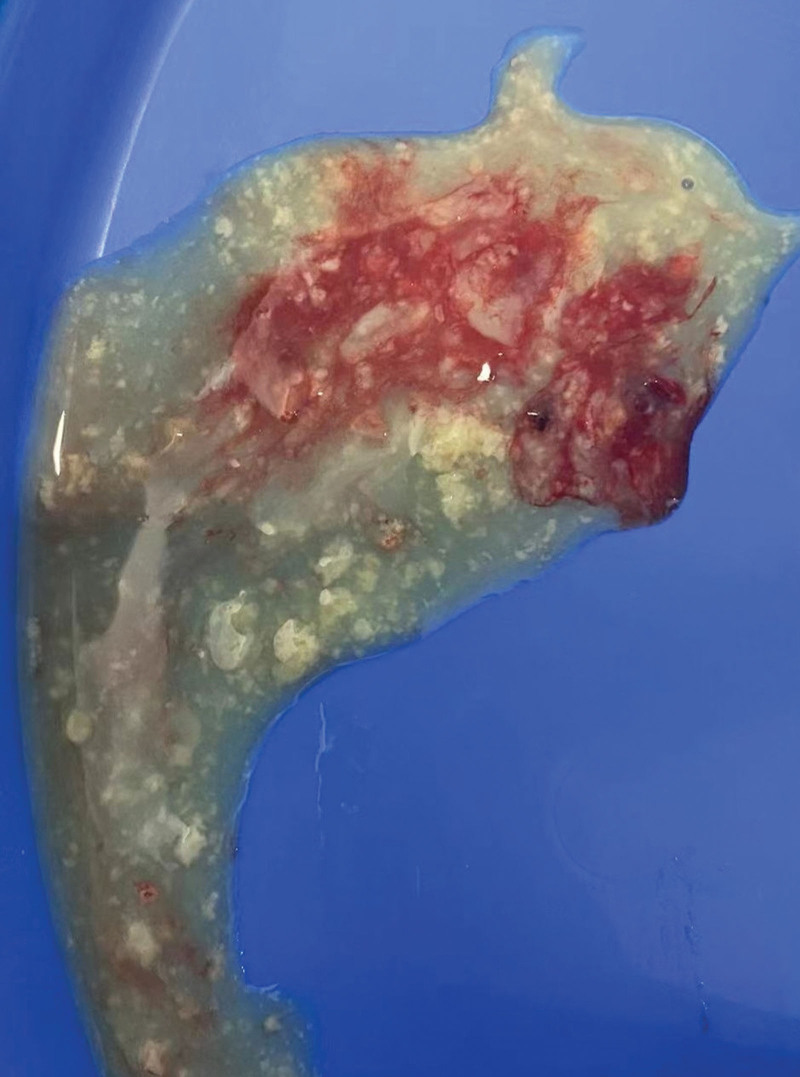
Cheese-like liquid.

**Figure 7.**
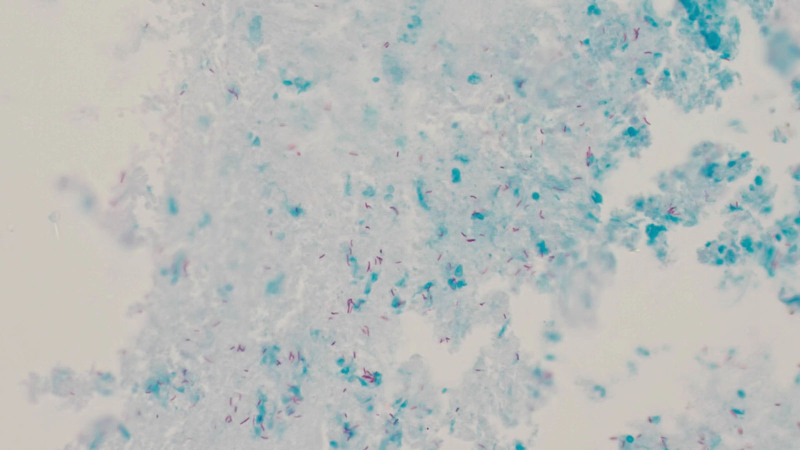
Histological examination revealed positive acid-fast staining, confirming bone tuberculosis.

The publication of this case report obtained written informed consent from the patient’s legal guardian. A 2-year-old female presented with right forearm swelling, particularly pronounced proximally, without significant pain, fever, night sweats, appetite loss, or weight loss. Her forearm, elbow, and finger movements were unimpaired, with no marked tenderness or percussion pain. The patient’s father had a history of treated pulmonary tuberculosis. Initial X-rays revealed extensive expansion and thinning of the right ulna’s lateral cortex, suggesting potential bone fibrous dysplasia or a cyst (Fig. 1). After 1 month, the right elbow swelling worsened (Fig. 2). Color Doppler ultrasound revealed lesions and soft tissue abscess formation in the right forearm bone (Fig. 3). Concurrently, CT scans demonstrated extensive expansion in the right ulna, featuring a lesion approximately 93 mm in length, along with evidence of bone destruction and cortical thinning (Fig. 4). Comprehensive X-ray examinations of the chest, pelvis, bilateral tibia and fibula, bilateral humerus, bilateral femur, left ulna, and radius showed no abnormal bone signs. Brain MRI results were normal. Blood tests indicated Hemoglobin: 67g/L; Leukocyte: 15.78*109/L; C-reactive protein: 93.62mg/L; and an erythrocyte sedimentation rate of 42 mm/H. The quantitative test for Mycobacterium tuberculosis DNA was negative, while T cell spot test for Mycobacterium tuberculosis and the tuberculin test were strongly positive (Fig. 5). Physical examination revealed swollen and deformed right forearm with noticeable swelling at the ulna’s proximal end and a fluctuant cold swelling. There was no skin flushing or fever, limited forearm rotation, normal finger movement, and no numbness. The patient was diagnosed with tuberculosis infection. During surgery under general anesthesia, a 0.5 cm incision was made at the swollen proximal right ulna, releasing 20 mL of a cheese-like liquid (Fig. 6). The wound was extensively irrigated with saline until clear fluid was obtained. A drainage tube was inserted, the incision closed, and the arm immobilized with an L-shaped plaster cast. One week postoperatively, the drainage tube showed no fluid outflow and was removed. Cultures from the tissue showed no bacterial growth or Mycobacterium tuberculosis. Pathological examination confirmed bone tuberculosis, with positive acid-fast staining (Fig. 7). The incision healed within 1 month. Postoperative anti-tuberculosis treatment included pyrazinamide, isoniazid, rifampicin, and ethambutol for 3 months, followed by isoniazid and rifampicin for 9 months.

## 3. Results

**Figure 8.**
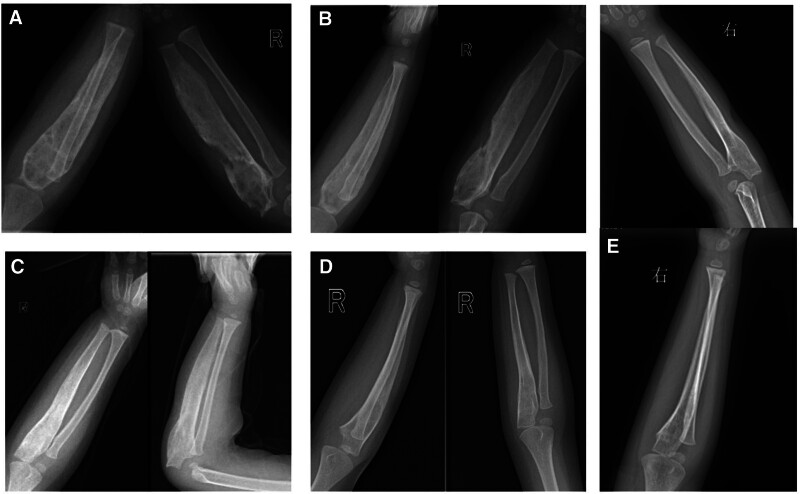
(A) At 2 years and 2 months old, X-ray imaging revealed extensive bone expansion in the ulna, with thinned cortical bone and disorganized trabecular structure. (B) At 2 years and 3 months old, X-ray indicated the ulna remained expanded with thin cortical bone, yet there was an increase in bone density. (C) At 2 years and 6 months old, X-ray imaging demonstrated a significant reduction in ulnar expansion, thickening of the cortical bone, and enhanced bone density. (D) At 2 years and 10 months old, X-ray showed the ulna’s expansion had resolved, with the bone’s shape nearing normal, normal cortical bone, and normal bone mineral density. (E) At 3 years and 6 months old, X-ray imaging confirmed normal ulnar morphology and bone structure, with no signs of recurrent bone tuberculosis or bone destruction.

The latest follow-up was conducted when the patient was 3 years and 6 months old. At that time, the patient did not exhibit any symptoms of lung disease, such as coughing and sputum production. Anti-tuberculosis treatment often causes liver dysfunction, by testing blood routine and liver function, they were found to be normal and corrected anemia. There was no apparent pain or discomfort in the limbs, and the swelling of the forearm had disappeared, returning to its normal appearance. Additionally, the rotation function of the forearm had returned to normal. X-ray examination revealed a gradual improvement in ulna dilatation, ultimately returning to its normal long tube bone shape without any signs of bone destruction (Fig. 8).

## 4. Discussion

Tuberculosis, an ancient disease caused by Mycobacterium tuberculosis, predominantly affects the lungs. Following pulmonary infection, tuberculosis can disseminate to the skeletal system and pose a risk to close contacts. In this case, the child’s infection may have originated from her father, who had tuberculosis a year prior. Extrapulmonary tuberculosis typically arises from lymphatic or hematogenous spread during primary infection, but occasionally, it presents independently without pulmonary involvement.^[1]^ Literature indicates that musculoskeletal tuberculosis accounts for 3 to 19% of cases, with the spine being the most frequently involved site, succeeded by the hip and knee joints.^[1,2]^ Bone tuberculosis is rare within the spectrum of tuberculosis diseases, with Mycobacterium tuberculosis preferentially infecting regions with high vascularity, such as the metaphyses of long tubular bones like femur and tibia. The upper limbs, being non-weight-bearing, have a lower incidence of tuberculosis, with UBT being exceptionally rare and infrequently reported in the literature.

Typical symptoms of bone tuberculosis include localized pain and swelling, often accompanied by systemic signs like low-grade fever, night sweats, appetite loss, and weight loss, which are hallmark clinical features of musculoskeletal tuberculosis.^[3,4]^ However, in this case, the patient exhibited none of these symptoms except for elbow swelling, characterized by a cold abscess without signs of acute infection such as redness, warmth, or pain. The atypical presentation in this instance led to an initial oversight of bone tuberculosis. literature indicates that bone tuberculosis during childhood often manifests with subtle and atypical symptoms, such as mild local pain and soft tissue swelling, which may persist for a duration ranging from several days to months.^[5]^ Due to its uncommon presentation and lack of specific symptoms, long tubular bone tuberculosis is prone to misdiagnosis and delayed identification.

In this case, the ulnar lesion exhibited severe damage and expansion at the proximal end, likely initiating from this area with a rich blood supply and progressively affecting the ulna’s distal end. The duration of tuberculosis infection is typically prolonged. Reports suggest that trauma might play a pivotal role in the pathogenesis of bone tuberculosis, with the lesion remaining asymptomatic until triggered by minor trauma.^[6–8]^ The patient’s timeline, inferred from the father’s account, suggests an illness duration exceeding 6 months.

Tuberculous osteomyelitis is characterized by 4 distinct radiographic features: transparency, infiltration, focal erosion, and the formation of a sequestrum.^[9]^ The radiographs in this case revealed extensive expansion of the right ulna, cortical thinning, and various high-density opacities, suggesting the possibility of bone fibrous dysplasia or a cyst. The presence of soft tissue edema also raised concerns about potential malignancy. Differential diagnoses include enchondroma, eosinophilic granuloma, giant cell tumor, aneurysmal bone cyst, and osteoblastoma, which can all manifest with long tubular bone expansion. The diagnosis of tuberculosis was considered following the development of a cold abscess. The affected ulnar segment included the metaphysis, showing expansive changes with the bone marrow cavity’s diameter approximately 3 times that of a normal ulna, a feature not typically seen in general infections. According to Watts, the radiographic hallmarks of tuberculosis are nonspecific and include bone destruction, soft tissue swelling, and mild periosteal reactions, with scarce necrotic bone and infrequent pathological fractures.^[10]^ Tuberculous osteomyelitis shows similar characteristics to Ewing sarcoma or fungal osteomyelitis and may be confused with cartilaginous tumors or Langerhans cell histiocytosis.^[11,12]^ These ambiguous radiographic signs contribute to the challenges of early diagnosis.

During the initial visit, the patient exhibited no pain or discomfort, and X-rays revealed no pathological fractures, leading to a recommendation for a pathological examination to confirm the diagnosis. The patient’s family opted for observation. A month later, the development of a cold abscess raised suspicions of tuberculosis. Subsequent tests revealed severe anemia, increased white blood cell count, and increased ESR. A study from Qatar indicated that 67% of pediatric tuberculosis cases presented with elevated ESR.^[13]^ The definitive diagnosis of bone tuberculosis relies on isolating Mycobacterium tuberculosis from the bone,^[10,14]^ or through positive histological findings or culture.^[3]^ In this case, extensive caseous necrotic tissue was extracted from the forearm, revealing granulomas, positive acid-fast bacilli staining, a positive tuberculosis T cell test, and a strongly positive tuberculin test, thereby confirming UBT. Mycobacterium tuberculosis was not isolated in this case.

The proximal ulna showed severe damage with the formation of caseous pus. Surgical intervention involving debridement and curettage of the affected tissue was employed. It is critical to ensure that the cystic cavity is interconnected and drainage is unobstructed during curettage. To comprehensively remove the lesion, a convection washing technique was utilized, inserting tubes at the ulna’s proximal and distal ends to facilitate the complete removal of the tuberculous tissue. Even if the epiphyseal plate is involved, complete debridement is essential to eradicate the infection, as the bone can potentially continue to grow normally despite epiphyseal plate damage.^[15]^ No bone grafting was performed to avoid the risk of dead bone formation, which could impede bone regeneration and increase the likelihood of nonunion in tuberculosis wounds. Dead bone formation might also lead to persistent sinus tract formation. The use of allogeneic bone could raise the risk of rejection. Many reports suggest that debridement alone, without bone grafting, yields favorable outcomes.^[16,17]^ However, the introduction of new bone graft materials, like calcium sulfate impregnated with anti-tuberculosis drugs, might offer improved results. Given the ulna’s expansive changes and cortical thinning, there is a risk of pathological fractures postoperatively, making the application of plaster fixation necessary for protection.

During the anti-tuberculosis treatment of patients, liver function and blood routine remained normal, without any signs of anemia. The patient’s appetite was stable, and there was a gradual increase in weight. After 12 months of treatment, the ulna’s bone destruction began to heal. Notably, the diameter of the expanded ulnar bone marrow cavity decreased, and the density of the trabecular bone increased, thus not necessitating an extension of the treatment duration. The concerns about permanent ulnar deformation and impaired forearm rotation, as well as the potential premature closure of the epiphysis, were not realized. After 18 months of follow-up, the ulna’s structure and the forearm’s functionality were normal. This recovery is attributed to the robust remodeling capacity of children’s long tubular bones, akin to the natural correction observed in children’s long tubular bone fractures. Continuous anti-tuberculosis treatment is crucial. Despite initial delays in diagnosis and treatment, the patient achieved a full recovery without any sequelae.

The limitation of this case is that the diseased tissue did not culture mycobacterium tuberculosis, which challenges our diagnosis of bone tuberculosis. It is feasible to diagnose bone tuberculosis by positive acid-fast staining, positive TSPOT test for Mycobacterium tuberculosis, and positive tuberculin test. However, the number of ulnar tuberculosis cases is small, and this kind of ulnar tuberculosis can not be used as a basis for the diagnosis of bone tuberculosis, but only as a reference symptom.

## 5. Conclusions

This case shows that tuberculosis can significantly enlarge the long tubular bone marrow cavity and thin the cortical bone, impacting the entire bone. However, comprehensive surgical debridement and consistent anti-tuberculosis treatment led to complete normalization of the bone’s structure and function, demonstrating a positive prognosis for extensive long tubular bone lesions caused by tuberculosis.

## Author contributions

**Data curation:** Qineng Mo, Yunlong Li, Chunli Ling.

**Formal analysis:** Xiaohua Wei, Xiansheng Xia, Guoxin Nan.

**Investigation:** Qineng Mo, Yunlong Li, Chunli Ling.

**Methodology:** Xiaohua Wei, Xiansheng Xia, Guoxin Nan.

**Resources:** Qineng Mo.

**Supervision:** Xiansheng Xia, Chunli Ling.

**Validation:** Guoxin Nan.

**Writing – original draft:** Qineng Mo.

**Writing – review & editing:** Qineng Mo.

## References

[1] Pai M Behr MA Dowdy D. Tuberculosis. Nat Rev Dis Primers. 2016;2:16076. 27784885 10.1038/nrdp.2016.76

[2] Shah I Dani S Shetty NS Mehta R Nene A. Profile of osteoarticular tuberculosis in children. Indian J Tuberc. 2020;67:43–5. 32192616 10.1016/j.ijtb.2019.08.014

[3] Watts HG Lifeso RM. Tuberculosis of bones and joints. J Bone Joint Surg Am. 1996;78:288–98. 8609123 10.2106/00004623-199602000-00019

[4] Kotwal PP Khan SA. Tuberculosis of the hand: clinical presentation and functional outcome in 32 patients. J Bone Joint Surg Br. 2009;91:1054–7. 19651833 10.1302/0301-620X.91B8.22074

[5] Hosalkar HS Agrawal N Reddy S Sehgal K Fox EJ Hill RA. Skeletal tuberculosis in children in the Western world: 18 new cases with a review of the literature. J Child Orthop. 2009;3:319–24. 19543761 10.1007/s11832-009-0184-7 PMC2726868

[6] Goldblatt M Cremin BJ. Osteo-articular tuberculosis: its presentation in coloured races. Clin Radiol. 1978;29:669–77. 737957 10.1016/s0009-9260(78)80198-6

[7] Mortensson W Eklöf O Jorulf H. Radiologic aspects of BCG-osteomyelitis in infants and children. Acta Radiol Diagn (Stockh). 1976;17:845–55. 1016508 10.1177/028418517601700612

[8] Davidson PT Horowitz I. Skeletal tuberculosis. A review with patient presentations and discussion. Am J Med. 1970;48:77–84. 4906108 10.1016/0002-9343(70)90101-4

[9] Kritsaneepaiboon S Andres MM Tatco VR Lim CCQ Concepcion NDP. Extrapulmonary involvement in pediatric tuberculosis. Pediatr Radiol. 2017;47:1249–59. 29052770 10.1007/s00247-017-3867-0

[10] Sudarshan K. Current concepts review. Tuberculosis of bones and joints. J Bone Joint Surg Am. 1997;79:1891. 9409801

[11] Pigrau-Serrallach C Rodríguez-Pardo D. Bone and joint tuberculosis. Eur Spine J. 2013;22(Suppl 4):556–66. 10.1007/s00586-012-2331-y PMC3691411 22711012

[12] Mikawa T Miyoshi K Fujita K Hase R Hosokawa N. The Great Imitator; clavicular tuberculosis mimics a metastatic neoplasm. Kansenshogaku Zasshi. 2015;89:588–91. 26630791 10.11150/kansenshogakuzasshi.89.588

[13] Al-Marri MR Kirkpatrick MB. Erythrocyte sedimentation rate in childhood tuberculosis: is it still worthwhile? Int J Tuberc Lung Dis. 2000;4:237–9. 10751069

[14] Rigaud M Borkowsky W. Tuberculosis in children. Tuberculosis. 2004;25:609–24.

[15] Ohtera K Kura H Yamashita T Ohyama N. Long-term follow-up of tuberculosis of the proximal part of the tibia involving the growth plate. A case report. J Bone Joint Surg Am. 2007;89:399–403. 17272458 10.2106/JBJS.E.01314

[16] Erol B Topkar MO Basar H Caliskan E Okay E. Solitary cystic tuberculosis of the distal femur and proximal tibia in children. J Pediatr Orthop B. 2015;24:315–20. 26035351 10.1097/BPB.0000000000000193

[17] Kao HK Yang WE Shih HN Chang CH. Physeal change after tuberculous osteomyelitis of the long bone in children. Chang Gung Med J. 2010;33:453–60. 20804676

